# Repair of Celiomesenteric Trunk Aneurysm and Review of the Literature

**DOI:** 10.7759/cureus.35923

**Published:** 2023-03-09

**Authors:** Santh Prakash Lanka, Sukhwinder Johnny S Sandhu, Houssam Farres, Young Erben

**Affiliations:** 1 Division of Vascular and Endovascular Surgery, Mayo Clinic, Jacksonville, USA; 2 Department of Radiology, Mayo Clinic, Jacksonville, USA

**Keywords:** repair of celiomesenteric trunk, open repair of celiomesenteric trunk aneurysm, celiomesenteric trunk aneurysm repair, celiomesenteric trunk aneurysm, abdominal visceral aneurysm, celiomesenteric trunk

## Abstract

A celiomesenteric trunk (CMT) is a rare anatomic variant of a common origin for the celiac and superior mesenteric arteries. It is further a seldom occurrence to have aneurysmal dilatation of the CMT. Herein, we describe a patient with a CMT aneurysm and his open surgical repair. The open surgical repair included debranching from the right external iliac artery to the splenic and common origin of the superior mesenteric and common hepatic arteries using a bifurcated knitted graft. Postoperative recovery was unremarkable, and follow-up imaging demonstrated an excluded CMT aneurysm with excellent blood flow to the intra-abdominal organs through the bifurcated graft.

## Introduction

The celiac artery (CA) and superior mesenteric artery (SMA) usually are separate branches of the abdominal aorta. During embryologic development, a cleft separates the splenic artery (third) and superior mesenteric artery (fourth) roots of the dorsal abdominal aorta, leading to a separate CA and SMA, respectively. When this cleft does not form, it leads to the common origin of the celiomesenteric trunk (CMT). The incidence of CMT is rare, and it has been reported to be between 0.42% and 2.7% in cross-sectional imaging studies [1]. CMT aneurysms are exceedingly rare, and less than 50 such cases are described in the literature.

## Case presentation

**Figure 1:**
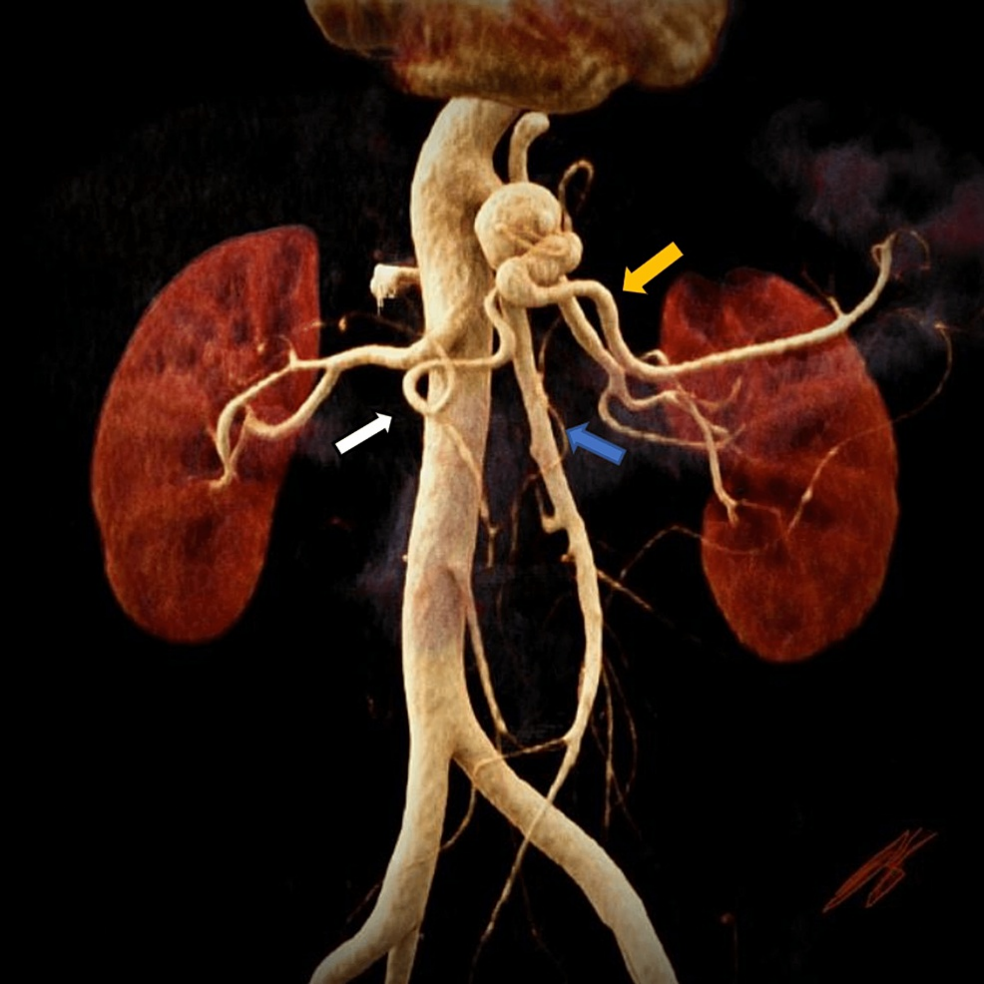
Pre-operative 3D reconstruction and cinematic rendering of the CTA with celiomesenteric trunk aneurysm denoting the splenic artery (orange arrow), hepatic artery (white arrow), and superior mesenteric artery (blue arrow) CTA: computed tomography angiography

A 68-year-old male patient was incidentally diagnosed with a CMT aneurysm during a routing gastroenterology examination 7 years before presentation. The aneurysm was followed annually, utilizing computed tomography angiography (CTA). In January 2023, it was found to be 23 mm in diameter (Figure 1).

**Figure 2:**
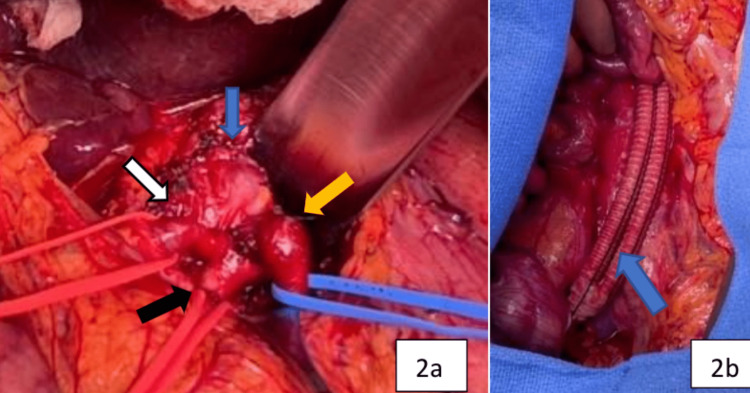
Intra-operative images (a) CMT aneurysm before repair (blue arrow) with vessel loops around the common hepatic artery (white arrow), superior mesenteric artery (black arrow), and splenic artery (orange arrow); (b) debranching bifurcated graft from the right external iliac artery to the splenic artery and the common origin of the common hepatic and superior mesenteric arteries CMT: celiomesenteric trunk

No other aneurysms or significant atherosclerotic disease was noted in the abdominal aorta or its branches. The patient’s past medical history included gastroesophageal reflux disease and renal calculi. Physical examination and pre-operative laboratory findings were unremarkable. From the operative perspective, the patient underwent a midline laparotomy, the transverse colon was retracted cephalad, and the small bowel was retracted laterally to the right of the patient. We then accessed the retroperitoneum by taking down the ligament of Treitz. We continued the exposure of the retroperitoneum distally to expose the aorta and the right common and external iliac arteries. We then proceeded to gain access to the lesser sac by traversing the gastrohepatic ligament. We then dissected free the splenic, superior mesenteric, and hepatic arteries. After heparinization, proximal and distal control of the external iliac artery was obtained. The debranching from this vessel to the splenic and common origin of the superior mesenteric and common hepatic arteries (Figure 2) was performed by utilizing a bifurcated knitted graft (Hemashield-Getinge, Göteborg, Sweden).

**Figure 3:**
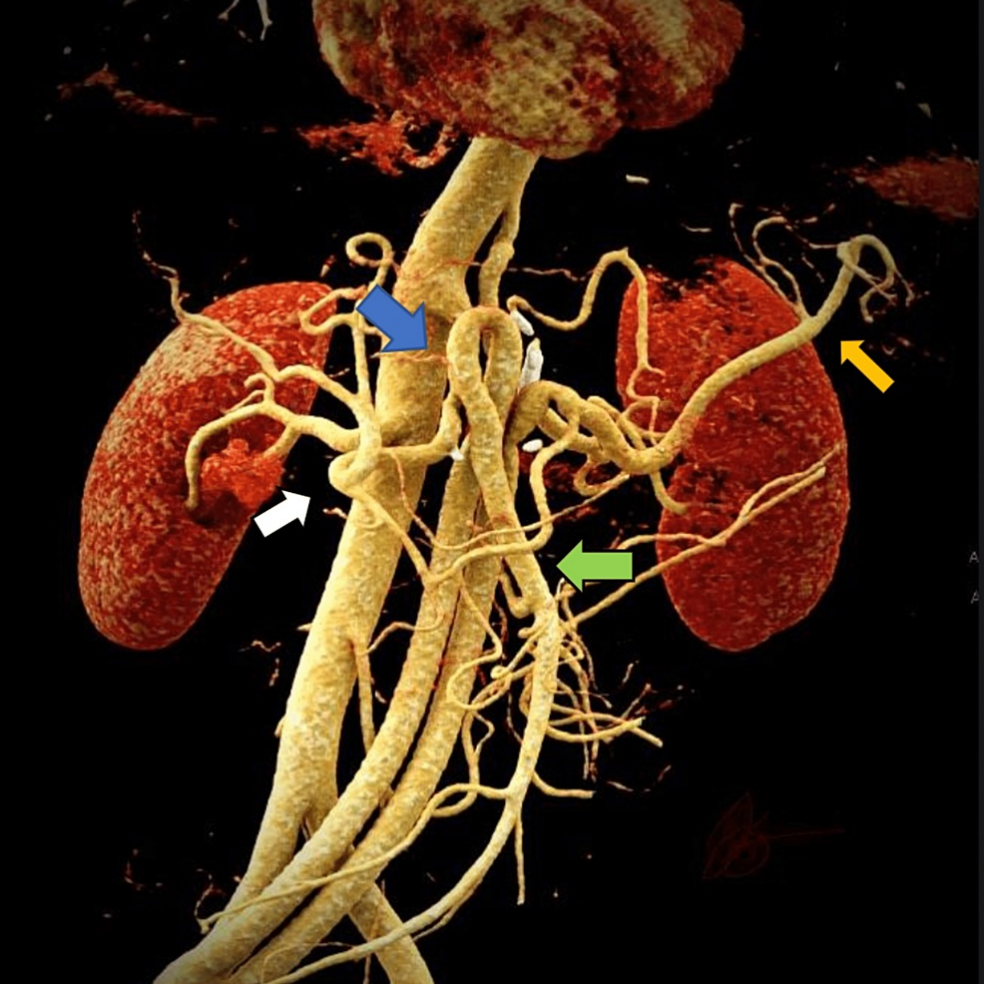
Postoperative 3D reconstruction and cinematic rendering of the CTA highlighting the debranching to the splenic artery (orange arrow) and the common origin (blue arrow) of the common hepatic (white arrow) and superior mesenteric (green arrow) arteries CTA: computed tomography angiography

The ischemia time for the right iliac, superior mesenteric/hepatic, and splenic arteries was 18, 20, and 11 min, respectively. The splenic artery was reconstructed to preserve the spleen function and the ease with which the exposure of this vessel was obtained. The anastomosis to the right external iliac (end to side) to the splenic (end to end) and en-bloc superior mesenteric and hepatic arteries (end to end) was performed using 4-0, 6-0, and 6-0 Prolene sutures (Surgo Surgical Supply, Ontario, Canada), respectively. The aneurysm was clipped proximally at its origin to the aorta, which decompressed the aneurysmal segment of the CMT. Because of the patient’s limited intra-abdominal fat and omentum, a bovine pericardium sheet was utilized to cover the graft and prevent direct contact with the knitted graft with the small intestine. Postoperatively, the patient had an excellent recovery. The total operative time was 254 min, with an estimated blood loss of 475 ml. He was started on a clear liquid diet on postoperative day (POD) 1 and on a general diet on POD 2. He worked with physical therapy on POD 2 and POD 3. CTA of the abdomen and pelvis was obtained on POD 2 (Figure 3), which demonstrated a bifurcated patent graft and complete collapse of the aneurysmal sac.

The patient was placed on Heparin 5000 IU as the anticoagulant of choice during the hospital stay for deep venous thrombosis prophylaxis and was discharged home with no long-term anticoagulant regimen. He was discharged from the hospital on POD 3 in excellent condition.

## Discussion

This case highlights a patient with a CMT aneurysm repaired in an open fashion using a debranching technique and proximal ligation of the inflow into the aneurysmal CMT. CMT aneurysms have a poor prognosis after rupture; hence, the Society for Vascular Surgery recommends elective repair when the aneurysm sac is > 2 cm in diameter, especially in women of childbearing age [2]. In our patient, an open surgical repair was preferred over an endovascular repair due to the patient’s young age and its complexity in terms of an endovascular repair with the significant risk of developing an endoleak that would preclude occlusion of the aneurysmal sac. Secondly, the choice for a retrograde inflow procedure was elected due to the high take-off of the left renal artery, which could have challenged the proximal clamping of the aorta (Figure 1).

Our literature review on CMT-related aneurysms revealed 21 case reports, including 29 patients (Table I) [3-20].

**Table 1:** Summary of the literature search AAA, abdominal aortic aneurysm; CA, celiac artery; CMT, celiomesenteric trunk; CT, celiac trunk; PTFE, polytetrafluoroethylene graft; SMA, superior mesenteric artery; SVG, saphenous vein graft

^Author (year)^	^Location of the aneurysm^	^Open/endovascular repair^	^Type of operation^	^Number of patients^
^Szczerepa et al. (2022) [3]^	^CMT aneurysm^	^Endovascular^	^Endovascular repair with main body extensions without coil embolization^	^1^
^Alam et al. (2020) [3]^	^SMA aneurysm^	^Endovascular^	^Endovascular coil embolization^	^1^
^Bi et al. (2020) [3]^	^CA aneurysm ^	^Endovascular^	^Endovascular bare metal stent-assisted coil^	^1^
^Oishi et al. (2020) [4]^	^CA aneurysm^	^Open^	^Aneurysmectomy and patch repair with SVG ^	^2^
^Lee et al. (2018) [3]^	^CA aneurysm^	^Endovascular^	^Endovascular coil embolization^	^1^
^VonDerHaar et al. (2015) [19]^	^CMT aneurysm with branches originating from the aneurysm^	^Open^	^Aneurysmectomy and vascular reconstruction (primary repair by joining the orifice of the SMA with the combined orifice of the common hepatic, splenic, and CMT)^	^1^
^Lipari et al. (2015) [18]^	^CA aneurysm^	^Open^	^Aneurysmectomy and vascular reconstruction (CT reimplanted onto the aorta)^	^1^
^Wang et al. (2014) [14]^	^CMT aneurysm^	^Open^	^Aneurysmectomy and vascular reconstruction (using trifurcated graft)^	^7^
^Higashiyama et al. (2011) [17]^	^CA aneurysm^	^Open^	^Aneurysmectomy and vascular reconstruction (common hepatic artery and splenic artery reimplanted onto the defect in the CMT)^	^1^
^Guntani et al.(2011) [16]^	^CA aneurysm ^	^Open^	^Aneurysmectomy and vascular reconstruction (branch of the CMT anastomosed with the common branch of the splenic artery and common hepatic artery)^	^1^
^Wang et al. (2010) [15]^	^CA aneurysm^	^Open^	^Aneurysmectomy and vascular reconstruction (primary repair and implantation of superior mesenteric artery onto SMA)^	^1^
^Lida et al. (2010) [20]^	^CA aneurysm^	^Open^	^Aneurysmectomy and vascular construction (anastomosing the splenic artery and common hepatic artery to the celiac trunk)^	^1^
^Obara et al. (2009) [13]^	^CMT aneurysm with AAA^	^Open^	^Aneurysmectomy and vascular reconstruction (open aorto bi-iliac limb placement with retrograde bypass from right iliac artery to the CA (end to side) and SMA (side to side)^	^1^
^Mammano et al. (2009) [12]^	^CMT aneurysm^	^Open^	^Aneurysmorrhaphy^	^1^
^Matsuda et al. (2006) [9]^	^CMT aneurysm^	^Open^	^Aneurysmectomy and vascular reconstruction (rejoining of the CT to the CMT)^	^1^
^Ailawadi et al. (2004) [5]^	^CMT aneurysm with supraceliac abdominal aneurysm^ ^CMT aneurysm with tortuous aorta^	^Open^	^Resection of the aortic aneurysm with a thoracoabdominal aortoaortic interposition graft, an aorto celiac artery Dacron bypass^ ^aortoaortic interposition PTFE graft to replace the tortuous upper abdominal aorta, a thoracic aorto-CME PTFE bypass graft ^	^2^
^Kalra et al. (2003) [11]^	^CA aneurysm^	^Open^	^Aneurysmectomy and vascular reconstruction (reimplantation of the celiac artery onto the CMT)^	^1^
^Detroux et al. (1998) [8]^	^CMT aneurysm^	^Open^	^Aneurysmorrhaphy^	^1^
^Matsumoto et al. (1996) [10]^	^CMT aneurysm^	^Open^	^Aneurysmectomy and vascular reconstruction (interposition graft with splenic artery implanted on it)^	^1^
^Bailey et al. (1991) [7]^	^CMT aneurysm^	^Open^	^Aneurysmorrhaphy with Dacron patch repair^	^1^
^Stanley et al. (1970) [6]^	^CMT aneurysm^	^Open^	^Aneurysmectomy and vascular reconstruction^	^1^

Four patients underwent endovascular repair [3], while the rest had an open repair [4-20]. The typical open reconstruction included aneurysmectomy with vascular reconstruction in 20 [5-6,9-11,13-20] aneurysmorrhaphy only in two [8,12] aneurysmorrhaphy with great saphenous vein patch in two patients [4] and Dacron patch in one patient [7]. There were two patients with a concomitant thoracoabdominal aneurysm, and both aneurysms were addressed in the same operation [5,13]. In all case reports, no postoperative complications were noted except for transient liver dysfunction reported by Matsumoto et al. [10]. Because of the variation in anatomy, the extreme rarity of this condition, and the high-risk nature of the operative intervention, it is vital to design an individualized care plan tailored to each patient’s specific characteristics and CMT aneurysmal anatomy.

## Conclusions

A CMT occurs due to the absence of a cleft formation during the embryologic development of the aorta. A CMT aneurysm is exceedingly rare, as evidenced by the limited number of reports in the literature. Therefore, an individualized operative plan is vital for a successful repair. In this case, we present a patient with a CMT aneurysm repaired using an open approach by debranching from the right external iliac artery and utilizing a bifurcated knitted graft to supply the splenic artery and the common origin of the hepatic and superior mesenteric arteries.
